# To see those not to be seen: cardiac uptake on noncardiac imaging

**DOI:** 10.1186/s44348-024-00024-3

**Published:** 2024-09-29

**Authors:** Sang-Geon Cho

**Affiliations:** Department of Nuclear Medicine, Chonnam National University Hospital, Gwangju, Republic of Korea

Since Perugini et al. [1] suggested a visual grading system based on the comparison of the cardiac uptake to surrounding bones, a series of studies repeatedly confirmed its values for diagnosing transthyretin cardiac amyloidosis (ATTR-CA), across different bone radiotracers [2, 3]. Now the diagnostic accuracy of bone scintigraphy reportedly reaches > 95% [4]. Although the image interpretation is so simple and does not require a complex analysis process, more advanced quantitative measurements are expected to further contribute to treatment response evaluation and prognostic stratification, which may not be effective with visual grading only [5, 6].

The excellent performances of bone scintigraphy in detecting ATTR-CA have drastically changed the clinical work-up process. Bone scintigraphy can diagnose ATTR-CA with excellent specificity even in the absence of histopathologic evidence of ATTR, across variable degrees of renal impairment [7]. Now the clinical guidelines invariably recommend nonbiopsy diagnostic criteria in patients with clinically suspected CA when serum/urine light chain assay is negative [8, 9]. Given the ability of accurate detection of ATTR-CA, some researchers have taken a look back on cardiac uptake, which is considered “not to be normally seen,” that might have been missed in daily non-cardiac bone scintigraphy reading. Throughout the systematic literature reviews, Treglia et al. [10] recently reported that the overall prevalence of “incidental” cardiac uptake suspicious for ATTR-CA ranges around 1.1% (95% confidence interval, 0.7%–1.4%), while the inter-report heterogeneity exists which probably reflects the differences in studies, patients, and index tests. Despite the low prevalence of cardiac uptake, the possibility of detecting ATTR-CA before clinical presentation would not be extremely rare among daily bone scintigraphy studies, considering the wide use of bone scintigraphy for various indications.

Navarro-Saez et al. [11] reported that older age is related to abnormal cardiac uptake on bone scintigraphy. Son et al. [12] further extends this relationship to a more specific diagnosis of ATTR-CA. The patients with unexplained diffuse cardiac uptake on bone scintigraphy was assigned as having “possible ATTR-CA,” and they were shown to have relatively older age and lower prevalence of end-stage renal disease.

However, it should be noted that 14 patients (61%) with cardiac uptake were assigned to possible ATTR-CA, while six of them did not undergo further evaluation for ATTR-CA. Only five had confirmative diagnosis of ATTR-CA while the others did not have plausible explanations for abnormal cardiac uptake. A recent study warns that such uptake actually predicts mortality among all-comers of bone scintigraphy, which should promote further evaluation [13]. It was found that even mild uptake (grade 1) could lead to worse prognosis and can progress to more intense uptake in the future. Thus, an immediate work-up should be provoked upon the detection of abnormal cardiac uptake even if the bone scintigraphy is not referred for an evaluation of cardiac pathology. The data from Son et al. [12] showed that the cross-sectional check of laboratory or echocardiographic indices might not help as they were not different between possible ATTR-CA versus non-CA; a longitudinal follow-up should be warranted in such patients.

It is quite interesting that bone scintigraphy additionally provides with diagnostic hints suggesting metastatic calcification involving the heart. A majority of non-CA patients with cardiac uptake had evidence of metastatic calcification [12]. Bone scintigraphy could visualize other sites of soft tissue calcification; single-photon emission computed tomography-computed tomography may further show the anatomic evidences of ectopic calcification, differentiating potential pitfalls from ATTR-CA.

In addition to the red-flag signs for ATTR-CA [14], the study suggests the demographic and image characteristics that should be kept in mind of the physicians interpreting bone scintigraphy [12]. However, the retrospective nature of the study indicates the need for a prospective study including thorough diagnostic work-up and prognosis data following the detection of abnormal cardiac uptake on bone scintigraphy.

## Data Availability

No datasets were generated or analysed during the current study.

## Abbreviation

ATTR-CA: Transthyretin cardiac amyloidosis
